# On the Origins of Terms in Binocular Vision

**DOI:** 10.1177/2041669521992381

**Published:** 2021-02-24

**Authors:** Nicholas J. Wade

**Affiliations:** Psychology, University of Dundee, Dundee, UK

**Keywords:** binocular terminology, horopter, stereoscope, stereoscopic vision, stereopsis, binocular rivalry, lustre, colour stereoscopy, anaglyph

## Abstract

Vision with two eyes has been commented upon for many centuries, and the principal concern has been with binocular single vision. The terminology we apply to binocular vision developed rapidly after the invention of the stereoscope in the early 19th century. The origins of terms such as anaglyph, binocular lustre, chromatic stereoscope, cyclopean eye, dichoptic, horopter, pseudoscope, rivalry, stereoscope, stereograph, and stereopsis are described together with portraits of those who introduced them.

## Introduction

**Figure 1.**
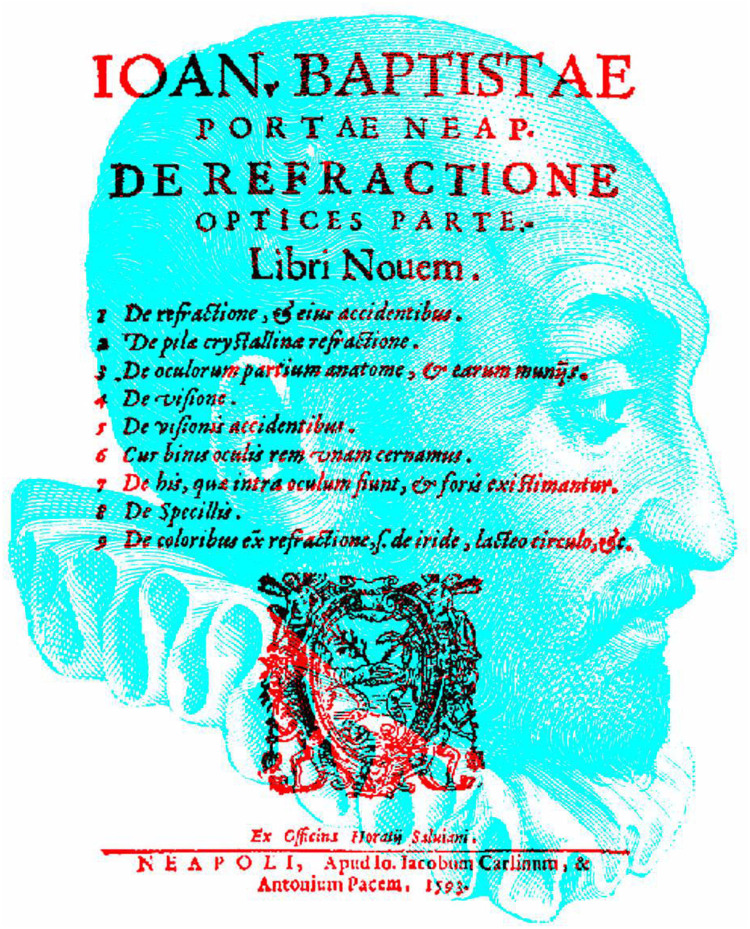
*Porta’s binocular vision* by Nicholas Wade. This and most of the subsequent images are displayed as anaglyphs to be viewed with red/cyan glasses so that the two components can be seen separately with different eyes. With the conventional red/left eye and cyan/right eye arrangement, the portraits will be seen by the left eye and the text/apparatus by the right eye.

Charles Wheatstone (1802–1875) wrote: “No question relating to vision has been so much debated as the cause of the single appearance of objects seen by both eyes” (1838, p. 387). Binocular single vision has been discussed at least since the time of Aristotle (384–322 BC), and, from 500 years later, it has been examined experimentally when Claudius Ptolemy (ca. 100–170) defined lines of visual correspondence for the two eyes (see Howard & Wade, 1996; Lejeune, 1989; Smith, 1996). Many of the early statements about binocular single vision are reflections of its breakdown and the experience of binocular double vision (Howard & Rogers, 1995; Wade, 1998). Clearly, vision with two eyes has been studied for many centuries, but the terminology that we use to describe it was transformed with the invention of the stereoscope by Wheatstone in 1832. Some terms relating to vision with two eyes were coined before that. For example, the term *binocular* can be found in a book on refraction by Giovanni Battista della Porta (1535–1615; 1593). His portrait is shown together with the title page of his book in Figure 1. It was concerned with optics, and the sixth book (on why we see one thing with two eyes) contains the Latin term *binos oculis*.

## Binocular Single Vision

**Figure 2.**
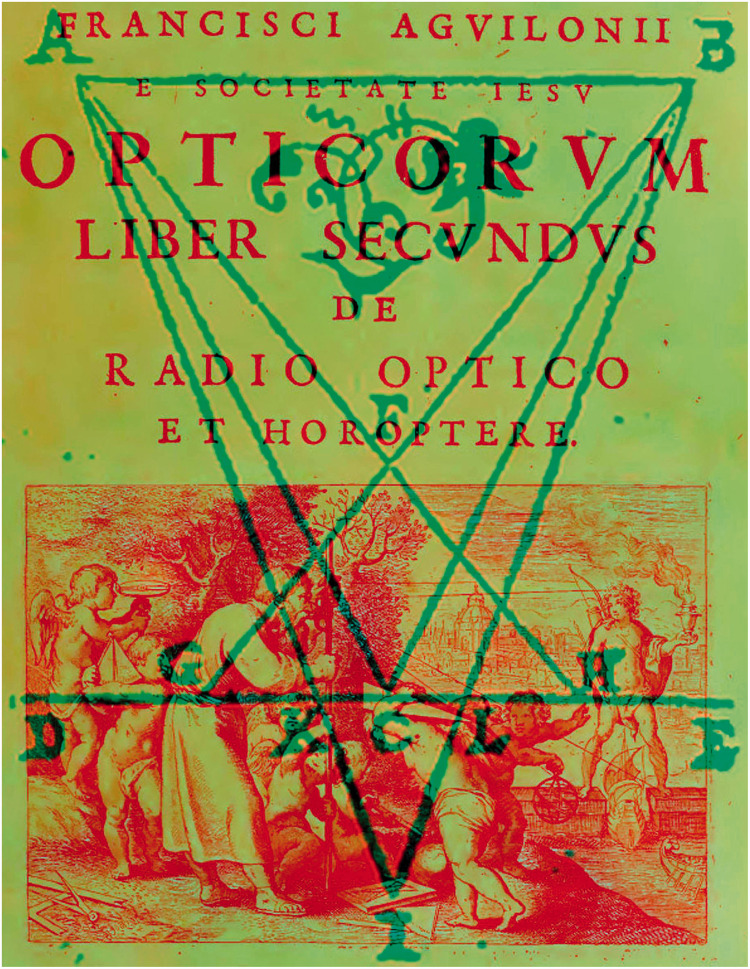
*Aguilonius’s horopter* by Nicholas Wade. Title page of Book II of *Optics* by Aguilonius (1613) together with his diagram of the horopter with fixation on the horopter plane (C) in front of it (F) and beyond it (I).

The overriding concern before the stereoscope was how the world is seen single with two eyes. Porta advanced a theory of binocular single vision that maintained we only see with one eye at one time. Two decades later, Franciscus Aguilonius (1567–1617; 1613) suggested an alternative interpretation—the images in the eyes are fused or combined. Some of the finest illustrations of the study of binocular vision are to be found in the book. The six frontispiece engravings were designed by his friend, Peter Paul Rubens (see Ziggelaar, 1983). The title page to Book II is shown in Figure 2. Aguilonius introduced the word *horopter* and also used the Latin term *stereographice* (stereographic); it was applied in the context of graphical representations of solid objects rather than for vision of solid objects. Stereographic projection was applied to geometry in the context of representing a sphere on a flat surface. Thomas Young (1773–1829) used it in this way in his lectures to the Royal Institution: “The stereographic projection of any circle of a sphere, seen from a point on its surface, on a plane perpendicular to the diameter passing through that point, is a circle” (1807, p. 22).

Aguilonius considered that the horopter was a flat plane, but this was challenged in the early 19th century when the horopter was shown to be a circle rather than a plane. First Charles Bell (1774–1842; 1803) and then Pierre Prevost (1751–1839; 1804) proposed that corresponding points fall on a circle passing through the point of bifixation and the centres of the eyes. This was formalised by Gerhard Ulrich Anton Vieth (1763–1836; 1818) and verified by Johannes Peter Müller (1801–1858; 1826). Wheatstone (1838) referred to it as a binocular circle, but it has become known as the Vieth–Müller circle. Both Vieth and Müller adopted the term *horopter* as introduced by Aguilonius. Müller later augmented his geometrical description of the circle of single vision by linking it with identical retinal points:

The horopter is therefore always a circle, of which the chord is formed by the distance between the eyes, or, more correctly, between the points of decussation of the rays of light in the eyes, and of which the size is determined by three points,—namely, by the two eyes, and the point to which their axes converge. (1838, 1843, p. 1196)

In this way, there were only two possible states of perception—single vision when objects fell on the circumference of the circle and double vision otherwise, and singleness was served by a fixed organic relation between nerve fibres. Thus, in the year that saw publication of Wheatstone’s article on stereoscopic depth perception, we find a statement by Müller denying its possibility.

## Stereoscopic Vision

**Figure 3.**
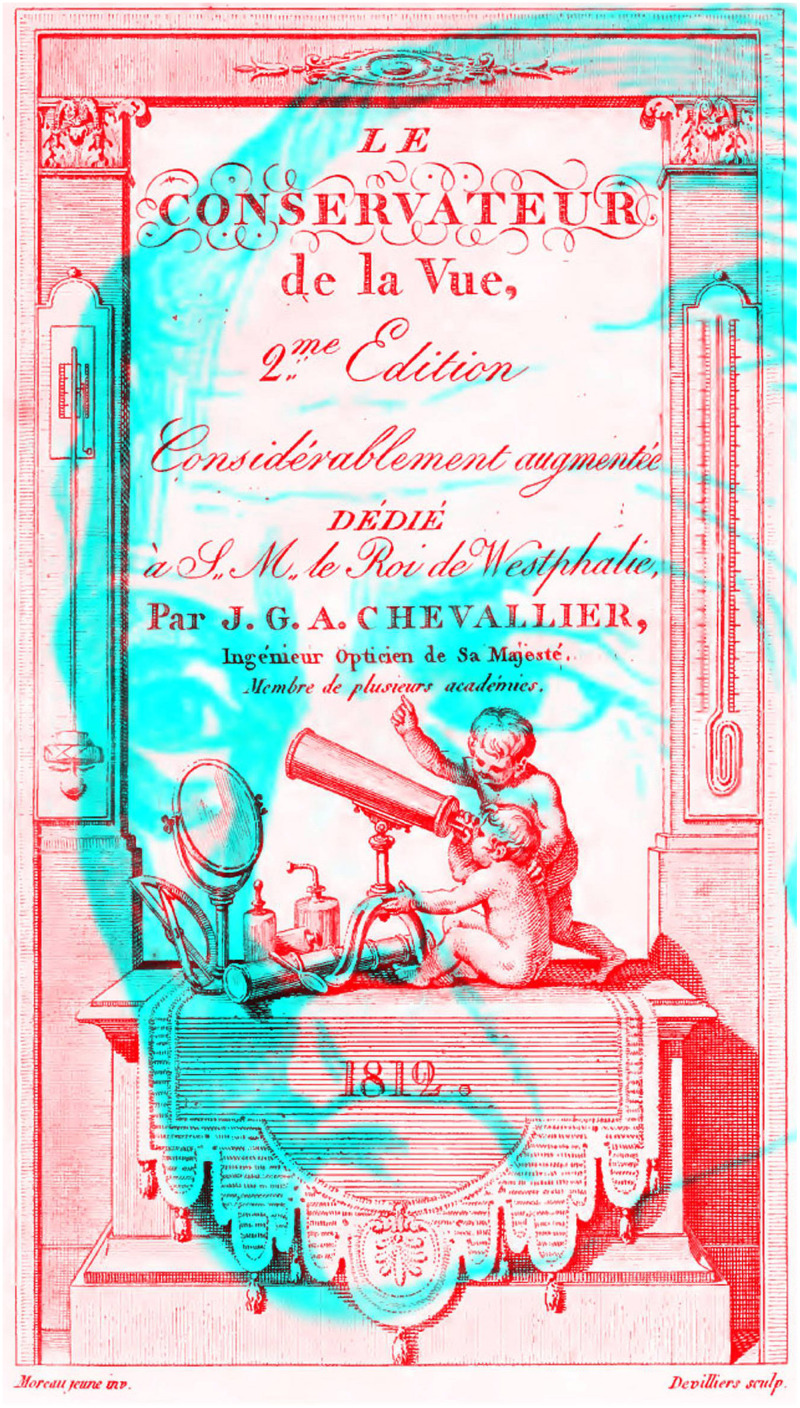
*Chevallier’s stéréoscope* by Nicholas Wade. Chevallier and the title page of his book in which a magic lantern device called a stéréoscope is described.

In 1812, Jean Gabriel Augustin Chevallier (1778–1848), a Parisian optical instrument maker, described an instrument he called a *stéréoscope*, but it was made for projecting two images from a magic lantern rather than for viewing with two eyes. Chevallier is shown in Figure 3 together with the title page of his book. The stéréoscope is described in the second edition of his book (Chevallier, 1812) but not the first (Chevallier, 1810). He was capitalising on the fashion for phantasmagoria that was sweeping Europe: Magic lantern slides of dramatic scenes were projected in all manner of locations and with special effects (like smoke) to create the feelings of fear and wonder in spectators (see Manonni, 2000).

**Figure 4.**
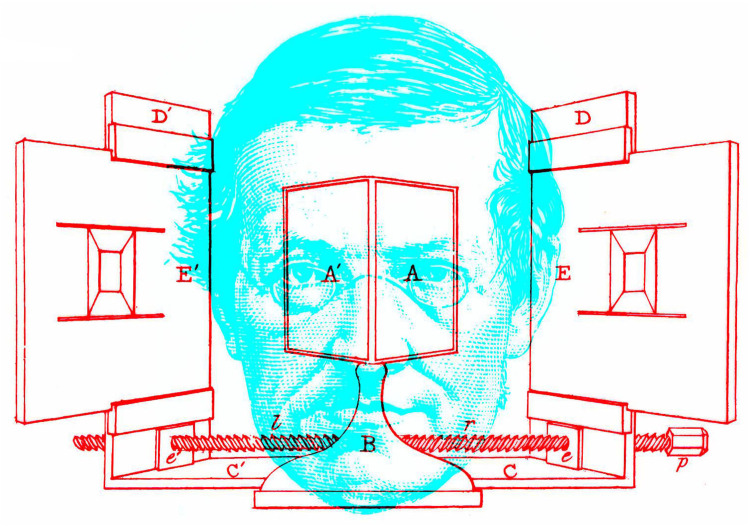
*Wheatstone’s mirror stereoscope* by Nicholas Wade.

What we now know as a *stereoscope* was invented by Wheatstone in the early 1830s, and he named it as such when he published his account of the instrument and his experiments with it. Following his discussion of the disadvantages of previous methods for combining images in two eyes, he stated “The frequent reference I shall have occasion to make to this instrument, will render it convenient to give it a specific name, I therefore propose that it be called a Stereoscope, to indicate its property of representing solid figures” (Wheatstone, 1838, p. 374). Wheatstone is shown in Figure 4 with his mirror stereoscope; in his memoir he illustrated only the reflecting stereoscope even though prism versions had been made for him in 1832 (see Wade, 1983). In his second memoir on binocular vision, Wheatstone (1852) referred to “new experiments relating to stereoscopic appearances” (p. 2).

Pseudoscopes reverse the disparities that normally exist so that concave objects appear convex or vice versa. It is as though the left and right eyes are being transposed. Wheatstone was well aware of the conversions of relief when reversing the cards in a stereoscope, but he was intrigued by the effects of reversing disparities when viewing three-dimensional objects. To achieve this, he described and named the *pseudoscope* in his 1852 memoir; it consisted of prisms. He applied it to reverse the normal relations between monocular and stereoscopic cues to depth: “With the pseudoscope we have a glance, as it were, into another visible world, in which external objects and our internal perceptions have no longer their habitual relation with each other” (Wheatstone, 1852, p. 12). He remarked on the difficulty of perceiving reversals of relief with the pseudoscope and the illuminating conditions that are necessary for such reversal.

William Oughter Lonie (1822–1894) was a teacher of mathematics at Madras College, St. Andrews, and in 1856, he was awarded the Prize Essay on the Stereoscope. In it he referred to the camera as “an instrument now indispensably requisite for stereoscopy” (Lonie, 1856, p. 13). The essay is fulsome in its praise of David Brewster (1781–1868) and his stereoscope, and it is hard to avoid the conclusion that this influenced Brewster in awarding the prize to Lonie. Nonetheless, Lonie does appear to have introduced the term *stereoscopy*.

**Figure 5.**
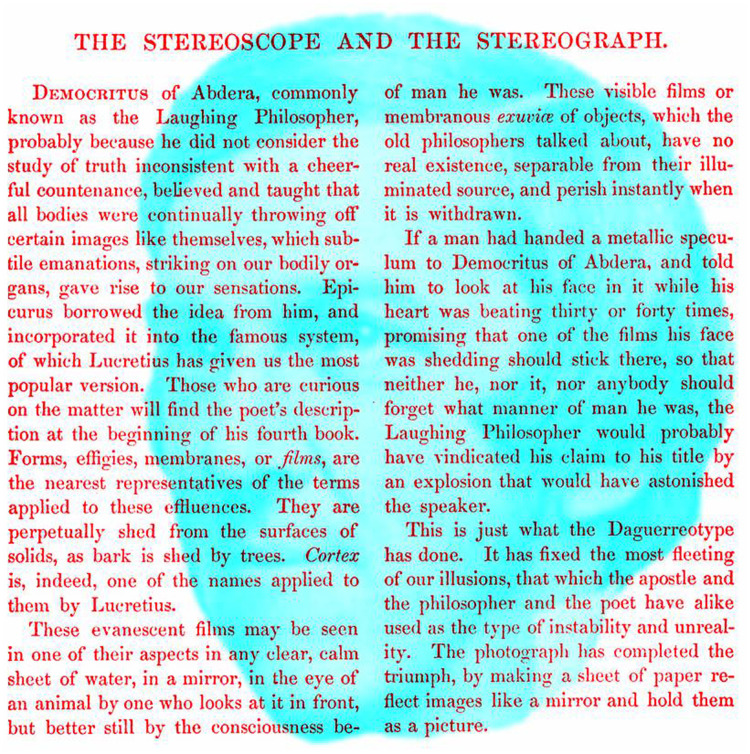
*Holmes’s stereograph* by Nicholas Wade.

The stereoscopic pictures themselves were not given a specific name by Wheatstone, and this want was supplied by Oliver Wendell Holmes (1809–1894; Figure 5). He wrote: “We have now obtained the double-eyed or twin pictures, or STEREOGRAPH, if we may coin a name” (1859, p. 743). Two years later, Holmes described his own design of stereoscope. It consisted of a pair of prisms mounted in a viewing case and an extending arm to which the cardholder for photographs could be placed and adjusted for viewing distance; it became known as the “American stereoscope” (Holmes, 1861, 1869, p. 1). 

The term *stereograph* is not now used as frequently as in the past, and it has tended to be replaced by *stereogram*. Hermann Ludwig Ferdinand Helmholtz (1821–1894) devoted many pages of his *Handbuch der physiologischen Optik* to stereoscopic vision. In the translation into English by James Powell Cocke Southall (1871–1962), there is reference to a stereogram (Helmholtz, 1925, p. 440), but the original German is “stereoscopic drawing” (stereoskopische Zeichnung; Helmholtz, 1867a, p.728). Similarly, in the French translation, it is referred to as a “dessin stéréoscopique” (Helmholtz, 1867b, p. 920).

**Figure 6.**
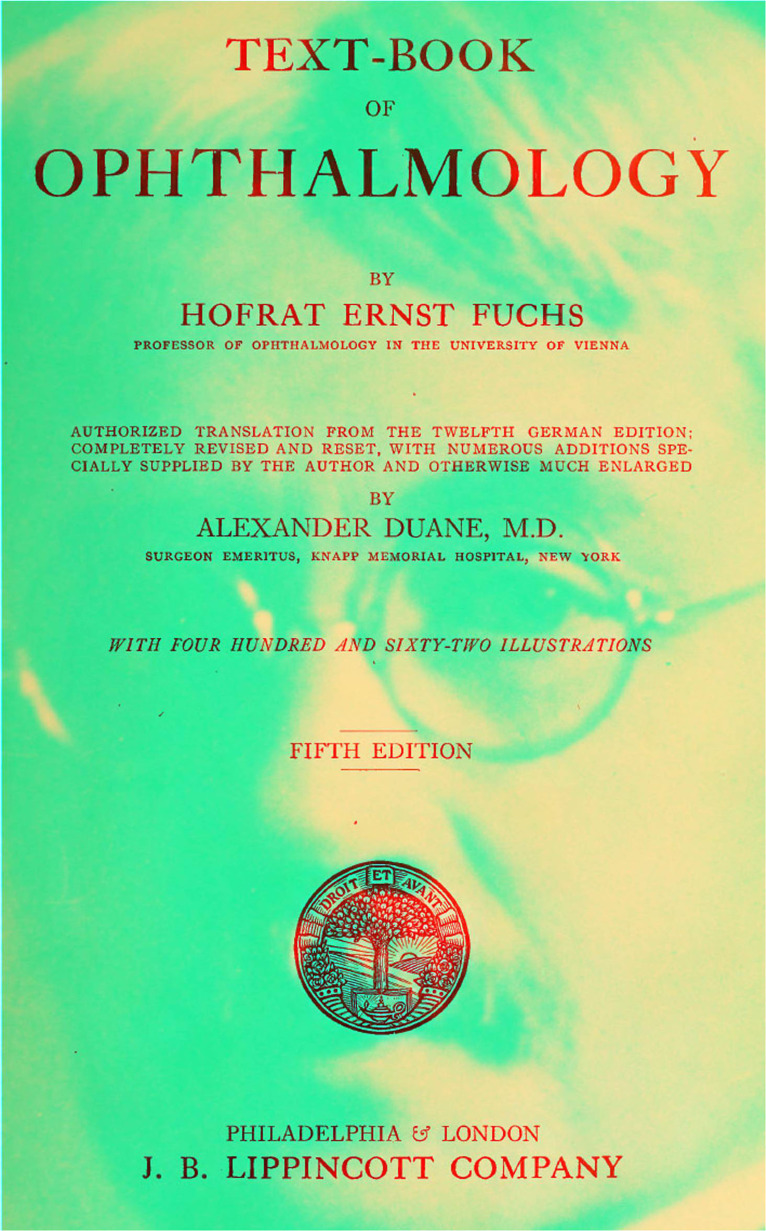
*Duane’s stereopsis* by Nicholas Wade.

*Stereopsis* as a shorthand for stereoscopic depth perception was used by the American ophthalmologist Alexander Duane (1858–1926; Figure 6) in 1917. Duane’s research interests were in accommodation and squint, and it is the latter that is relevant to stereoscopic depth perception. He translated Ernst Fuchs’s textbook of ophthalmology from German into English, and it is in the fifth edition that the term stereopsis is introduced: “The stereoscope and especially the amblyoscope will show both the patient’s ability to perform fusion and to secure stereoscopic vision (stereopsis)” (Duane, 1917, p. 773). The text was added by Duane, and it does not appear in earlier English editions of the book. Stereopsis was the term adopted by ophthalmologists associated with the Medical Research Laboratory at Mineola, New York, such as Howard, Dolman, Wilmer, and Verhoeff, and was widely used in America thereafter (see H. J. Howard, 1919; D. W. Wells, 1920). 

**Figure 7.**
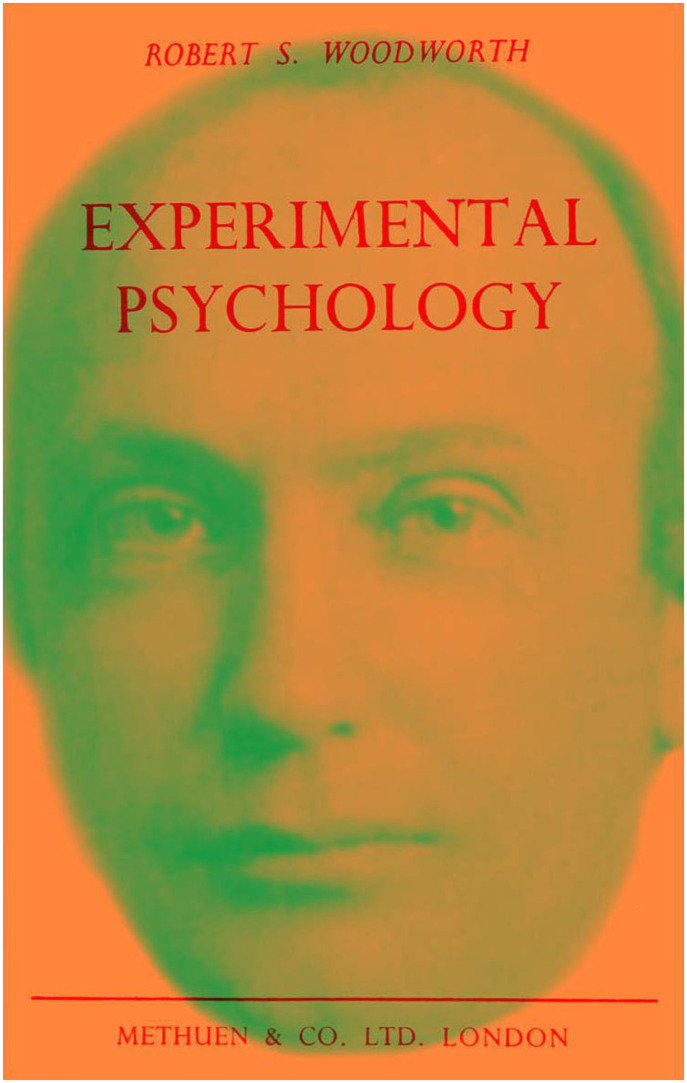
*Dichopic Woodworth* by Nicholas Wade.

The term *dichoptic* has not been used consistently since it was introduced by Robert Sessions Woodworth (1869–1962; Figure 7) in 1938 (although he used the word *dichopic*). When Woodworth was treating binocular vision in his *Experimental Psychology*, he wrote: “In a type of experiment which might be called *dichopic* (by analogy with dichotic and dichorhinic experiments in hearing and smell) discrepant stimulation is applied to corresponding parts of the two retinas” (Woodworth, 1938, p. 572). According to this definition, dichopic could apply to stereoscopic depth perception as well as to binocular rivalry. Woodworth’s dichopic gradually changed into the more widely used dichoptic (see Wade & Ono, 2005). Howard and Rogers (1995) distinguish between dioptic and dichoptic stimulation. The former refers to a single stimulus viewed with two eyes, whereas dichoptic stimuli are presented one to each eye, usually by means of a stereoscope. To differentiate dichoptic from stereoscopic stimulation, it can be restricted to those situations in which different but non-overlapping patterns are presented to each eye.

**Figure 8.**
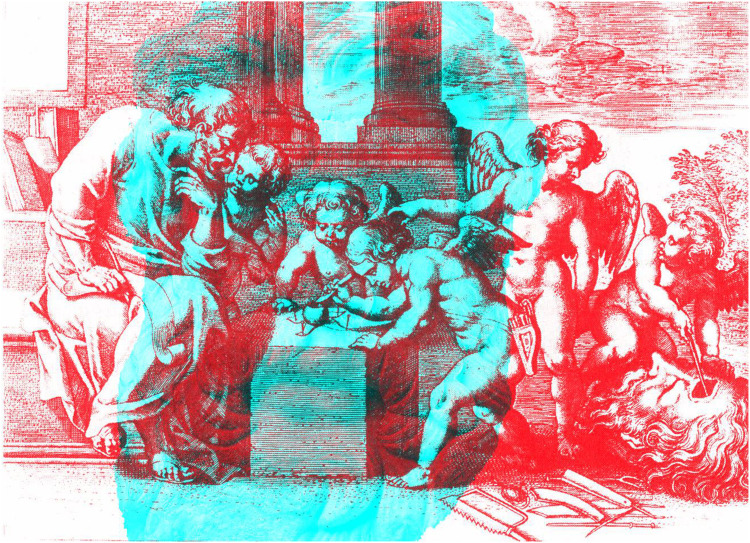
*Putti dissecting the eye of cyclops* by Nicholas Wade.

The concept of the *cyclopean eye* is probably embodied in the mythological cyclops who forged thunderbolts for Zeus; in the Homeric *Odyssey* cyclops was a one-eyed giant. The location of the single eye was central in the forehead, and the locus of binocular visual direction is now referred to as the cyclopean eye. The illustration by Rubens for Book I of Aguilonius’s book on optics (Figure 8) shows putti performing an operation beyond the scope of modern medicine—the cyclopean eye is being dissected. In the background, the one-eyed giant Polyphemus looks on.

With both eyes fixating on an object, it will be seen as single and in a direction corresponding to an origin between the eyes. The concept was given empirical support by William Charles Wells (1757–1817; 1792) in his book on single vision with two eyes (see Wade, 2003): An object is seen on a common axis from it to a point between the eyes. However, Wells did not give the origin of the common axis a name; this was supplied about 70 years later. Joseph Towne (1806–1879) was one of the few who cited Wells and conducted many experiments in stereoscopic vision (see Wade et al., 2006). He wrote: “that we see in the direction of the median plane of the head as from one central eye” (Towne, 1866, p. 301).

**Figure 9.**
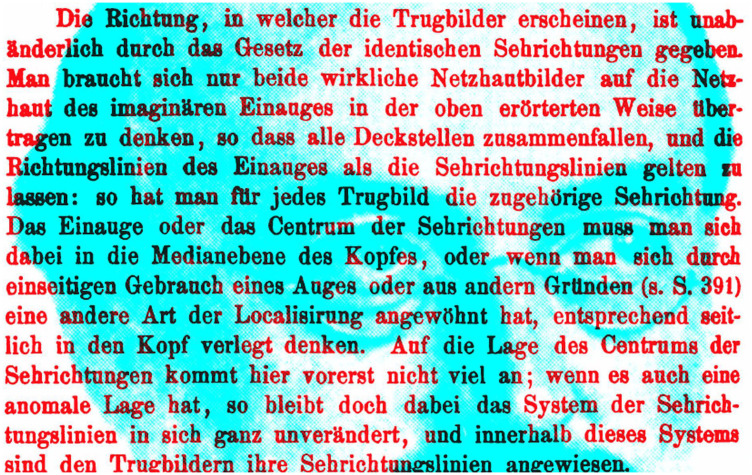
*Hering’s imaginary single eye* by Nicholas Wade.

Ewald Hering (1834–1916; 1879; Figure 9) provided the experimental confirmation of Wells’s research and he referred to an imaginary single eye between the two anatomical eyes:

The direction in which the illusional images appear, is unalterably determined by the law of identical visual directions. If we conceive of the two retinal images as transferred to the retina of the imaginary single eye (cyclopean), in a manner to make all cover points coincident, and let the lines of direction of the single eye pass for the visual lines of direction: each illusional image will have its own visual direction. One must imagine the single eye, or center of visual direction, as lying in the median plane of the head. (Hering, 1942, p. 74)

Figure 9 shows the eyes of Hering (1879) and the German text from his book on spatial vision describing an imaginary single eye (p. 426).

Hering essentially rediscovered the principles of visual direction described by W. C. Wells, although no reference was made to Wells’s earlier enquiries (Ono, 1981). The word *cyclopean* was added in Radde’s translation, as it was not present in Hering’s original text in which he referred to it as “an imaginary single eye” (see Figure 9). The term is now in common usage, and it was introduced in this context by Helmholtz; the German “Cyclopenauge” becomes the English “cyclopean eye”:

Midway between the two eyes suppose there were an imaginary cyclopean eye which was directed to the common point of fixation of the two eyes, and that it rolled according to the law governing the rolling of the two real eyes. Imagine the retinal images transferred from one of the real eyes to this imaginary eye, so that the point of fixation of the imaginary eye is the same as that of the real eye. *Then the points of the retinal image will be projected out along the line of direction of the imaginary cyclopean eye*. (Helmholtz, 1925, p. 258)

**Figure 10.**
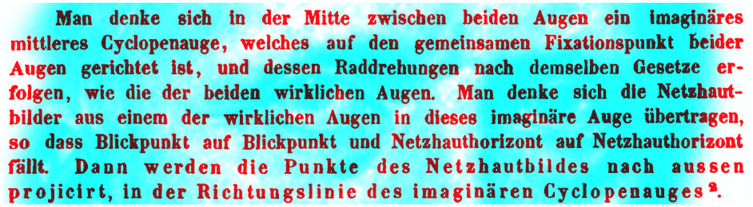
*The “Cyclopenauge” of Helmholtz* by Nicholas Wade.

The eyes of Helmholtz are shown in Figure 10 together with the German text referring to a cyclopean eye (1867a, p. 611).

Modern usage of the concept of the cyclopean eye does not necessarily correspond to that applied by Helmholtz. The term cyclopean vision is now synonymous with some representation in the brain that is combined from the two eyes (Julesz, 1971). Alternative terms for the conceptual central eye are binoculus and egocentre.

## Binocular Rivalry and Lustre

Binocular rivalry has been studied for longer than stereoscopic depth perception (see Wade, 1998; Wade & Ngo, 2013). Porta (1593) described the fluctuating visibility that accompanies viewing radically different patterns in each eye:

Nature has given us two eyes, one on the right and the other on the left, so that if we are to see something on the right we use the right eye, and on the left the left eye. It follows that we always see with one eye, even if we think both are open and that we see with both. We may prove it by these arguments: To separate the two eyes, let us place a book before the right eye and read it; then someone shows another book to the left eye, it is impossible to read it or even see the pages, unless for a short moment of time the power of seeing is taken from the right eye and borrowed by the left. (pp. 142–143)

The term *rivalry* entered into use in the mid-19th century. In his classic article, Wheatstone (1838) also described the fluctuating appearances of the letters A and S when presented to corresponding regions of the two eyes, but he did not assign a name to the ensuing perception. When Brewster (1844) addressed the same issue he called it *ocular equivocation*, a description that was not widely adopted (see Wade, 2019). Brewster (1844) wrote: “The *ocular equivocation*, as it may be called, which is produced by the capricious disappearance and reappearance of images formed on nearly corresponding points of each eye, is placed beyond a doubt by Mr Wheatstone’s own experiments” (p. 359).

**Figure 11.**
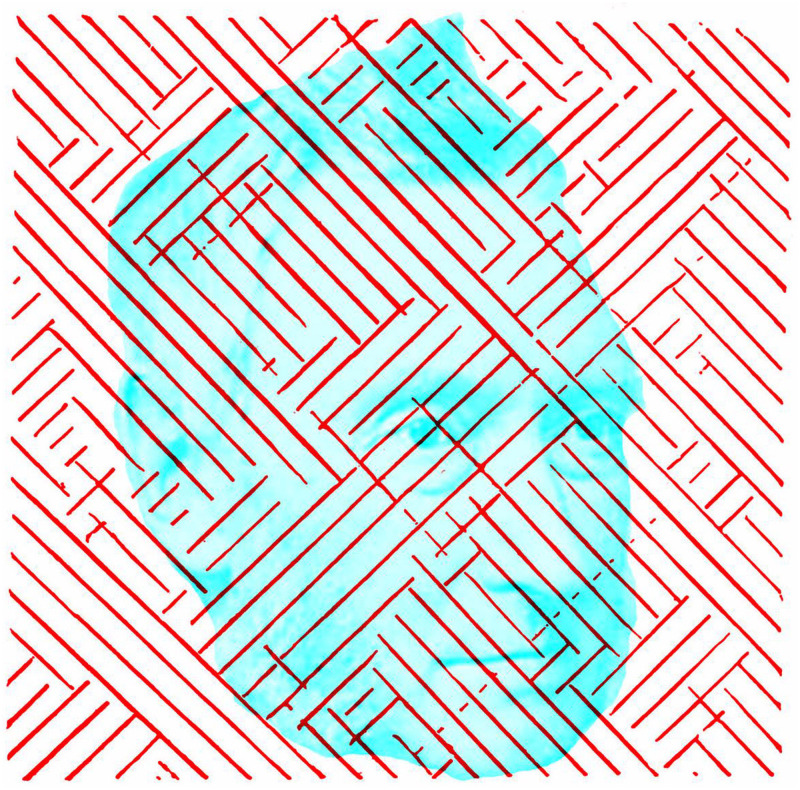
*Panum’s rivalry* by Nicholas Wade.

The term rivalry (“Wettstreit” in German) was used by Hermann Meyer (1815–1892; 1856) and by Peter Ludvig Panum (1820–1885; 1858) in his book on vision with two eyes; Panum’s precise words were “Wettstreit der Sehfelder” (rivalry of the visual fields). He recognised that letter shapes were complex patterns and simpler stimuli were soon enlisted. Those Panum introduced have dominated the study of rivalry ever since—orthogonal gratings. Panum is shown in Figure 11 together with a representative illustration from his book of what is seen during rivalry. With regard to crossed gratings, he wrote:

The rivalry of contours is at its strongest if the different lines in the two images are as equal as possible with regard to thickness and light intensity… The resulting composite image in the joint visual field cannot easily be drawn due to its constant restless variation; at one moment the diagonal lines of one side appear alone, at another those of the other side, but mostly some lines of both stimuli are present, so that in one place the inclined lines from one predominate and in others those of the other places, and both are visible in some locations though weaker and similarly washed out or blurred. (Panum, 1858, p. 38)

**Figure 12.**
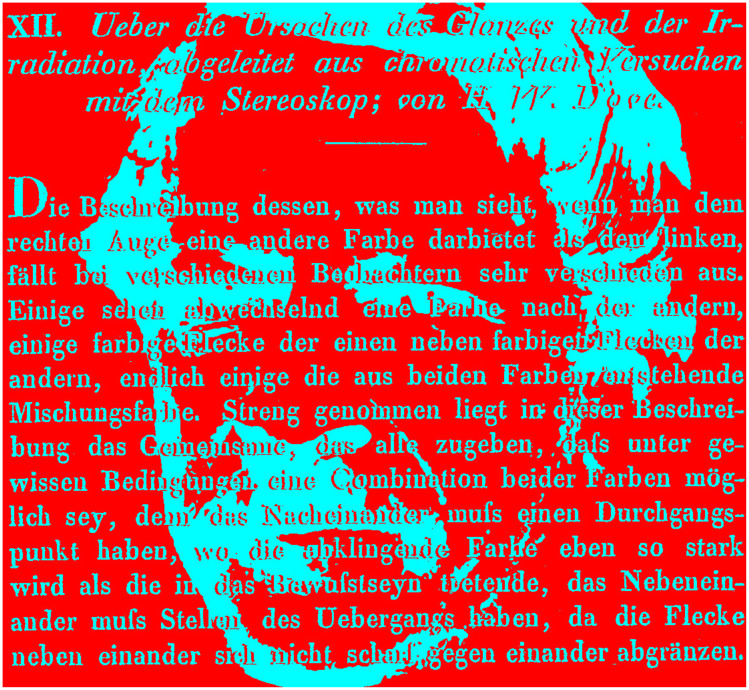
*Dove’s lustre* by Nicholas Wade.

*Binocular lustre* refers to the metallic impression created by combining positive and negative images (particularly when they are black on white and white on black) in the two eyes. The phenomenon was described by Heinrich Wilhelm Dove (1803–1879; 1851; Figure 12), and he called it gloss (“Glanz” in German). It was referred to as lustre in the English translation of Dove’s article (Dove, 1852). Brewster (1861) followed up Dove’s observations and called the phenomenon binocular lustre. Helmholtz (1867a, 1925) referred to it as stereoscopic lustre.

Dove wrote:

The projection for one eye was drawn in white lines upon a black ground, and for the other eye with black lines upon a white ground. A most remarkable result was obtained by the stereoscopic combination of both. The relief started into existence with surfaces which shone like graphite, having their edges formed of dazzling white and deep black lines which run parallel and in contact with each other throughout. (Dove, 1852, p. 242)

## Anaglyphs and the Chromatic Stereoscope

**Figure 13.**
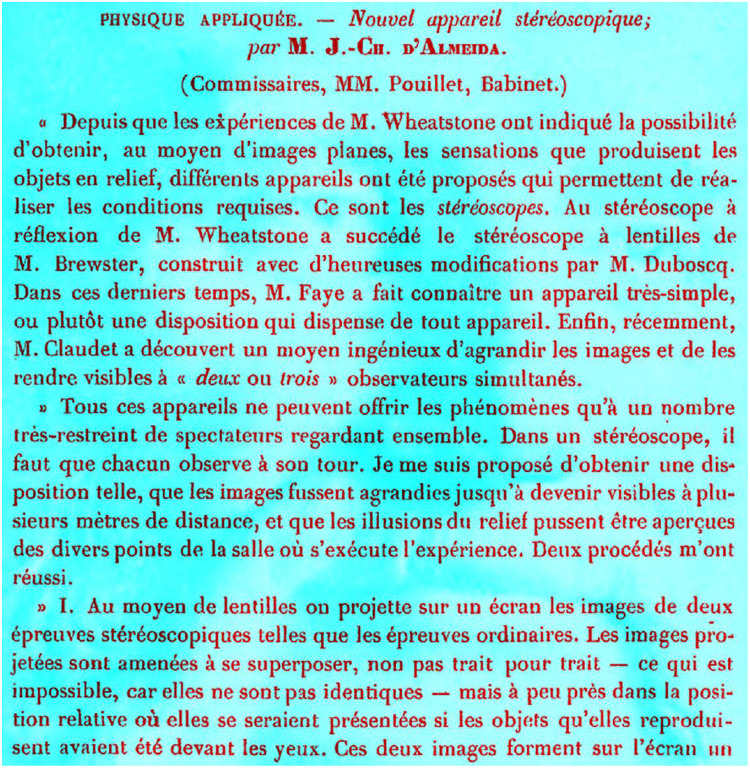
*D’Almeida’s colour separations* by Nicholas Wade.

The first stereoscopes were based on mirrors, prisms, or lenses. The use of colours for separating the eyes to see depth was realised by Wilhelm Rollmann (1853), a German inventor; the colours that he found worked best were blue and yellow drawings combined with red and blue glasses. Five years later, Joseph-Charles D’Almeida (1822–1880; Figure 13) described a similar system using images projected with two magic lanterns having colour filters in front of the lenses; the observer viewed the superimposed projections through corresponding filters (D’Almeida, 1858). He found that combinations of red and green projections and glasses worked well.

**Figure 14.**
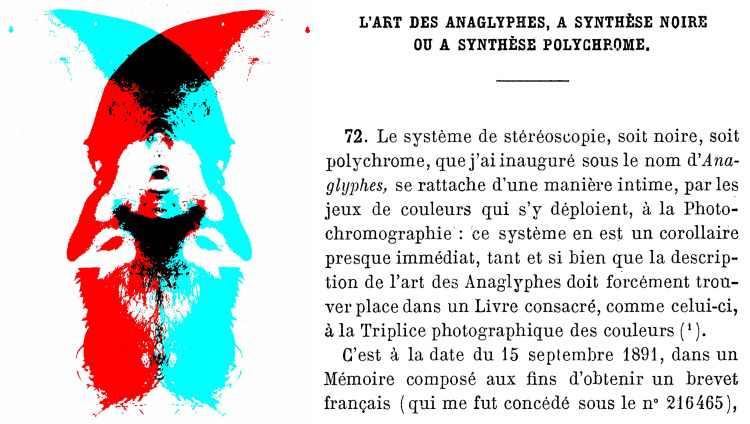
*Ducos Du Hauron’s anaglyph* by Nicholas Wade.

The proposals of Rollmann and D’Almeida had relatively little impact until Louis Ducos du Hauron (1837–1920) devised a method of overprinting red and blue or green designs, patented in 1891, to which the name *anaglyph* was given. Figure 14 shows a double portrait of Louis Ducos Du Hauron overprinted in red and cyan together with his text describing the date of the patent (from A. Ducos Du Hauron, 1897, p. 414). Thereafter, anaglyphs became increasingly popular as a means for printing and projecting stereoscopic drawings and photographs.

When long- and short-wave colours (such as red and blue) are placed near to one another, they can appear to occupy different depths. Usually red seems closer than blue but some people experience the opposite. It is called colour stereoscopy or chromostereopsis, and it has a long history in science and art. In his book on the theory of colours, Johann Wolfgang Goethe (1749–1832) wrote extensively about colour contrasts. He also made passing reference to differences in the apparent depth of colours:

In looking steadfastly at a perfectly yellow-red surface, the colour seems actually to penetrate the organ… a blue surface seems to retire from us… we love to contemplate blue, not because it advances to us, but because it draws us after it. (1810, pp. 294–295)

However, Goethe was referring to viewing large surfaces of a single colour rather than comparing the relative apparent distances of juxtaposed or adjacent colours which are the situations required for colour stereoscopy.

**Figure 15.**
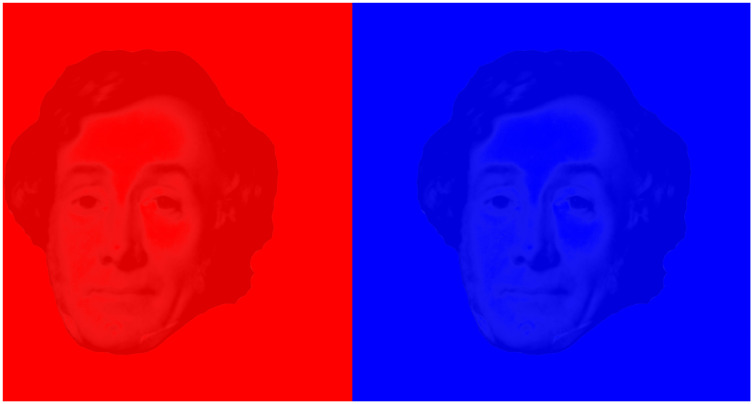
*Brewster’s colour distances* by Nicholas Wade.

A clear description of the association of colour and stereoscopic vision was provided by Brewster (1848) who is shown in Figure 15. He delivered four papers to the Mathematics and Physics Section of the British Association for the Advancement of Science at the 1848 meeting held at Swansea. One had the title “On the vision of distance as given by colour” in which he discussed the differences between the apparent distances of adjacent red and blue lines or surfaces:

When the boundary lines on a map are marked with two lines of different colours, the one rises above or is depressed below the other, and the two lines appear to be placed at different distances from the eye. This remarkable effect is most clearly seen when we look with both eyes through a large reading-glass, spectacles being used along with it by those who require them. (p. 48)

Brewster interpreted the colour-depth difference with the same concepts he adopted for stereoscopic depth perception. Apparent depth was determined by the point at which the two visual axes intersected; in the case of colour stereoscopy, the convergence of the visual axes for long and short wavelengths of light differed which he argued accounted for the difference in perceived depth. Brewster (1852) amplified his account a little and referred to the observation of different colours though a large lens as a chromatic stereoscope.

## Conclusion

Vision with two eyes involves them cooperating with one another to yield singleness and depth as well as competing which results in rivalry. The terminologies we apply to these aspects of vision have been greatly influenced by the phenomena exposed by the use of stereoscopes.
